# Genomes and secondary metabolomes of *Streptomyces* spp. isolated from *Leontopodium nivale* ssp. *alpinum*

**DOI:** 10.3389/fmicb.2024.1408479

**Published:** 2024-06-14

**Authors:** Fabian Malfent, Martin Zehl, Rasmus H. Kirkegaard, Martina Oberhofer, Sergey B. Zotchev

**Affiliations:** ^1^Division of Pharmacognosy, Department of Pharmaceutical Sciences, University of Vienna, Vienna, Austria; ^2^Vienna Doctoral School of Pharmaceutical, Nutritional and Sport Sciences (PhaNuSpo), University of Vienna, Vienna, Austria; ^3^Department of Analytical Chemistry, Faculty of Chemistry, University of Vienna, Vienna, Austria; ^4^Division of Microbial Ecology, Centre for Microbiology and Environmental Systems Science, University of Vienna, Vienna, Austria; ^5^Joint Microbiome Facility of the Medical University of Vienna and the University of Vienna, Vienna, Austria

**Keywords:** Edelweiss, endophytes, *Streptomyces*, genome mining, secondary metabolites

## Abstract

Bacterial endophytes dwelling in medicinal plants represent an as yet underexplored source of bioactive natural products with the potential to be developed into drugs against various human diseases. For the first time, several *Streptomyces* spp. were isolated from the rare and endangered traditional medicinal plant *Leontopodium nivale* ssp. *alpinum*, also known as Edelweiss. In the search for novel natural products, nine endophytic *Streptomyces* spp. from Edelweiss were investigated via genome sequencing and analysis, followed by fermentation in different media and investigation of secondary metabolomes. A total of 214 secondary metabolite biosynthetic gene clusters (BGCs), of which 35 are presumably unique, were identified by the bioinformatics tool antiSMASH in the genomes of these isolates. LC-MS analyses of the secondary metabolomes of these isolates revealed their potential to produce both known and presumably novel secondary metabolites, whereby most of the identified molecules could be linked to their cognate BGCs. This work sets the stage for further investigation of endophytic streptomycetes from Edelweiss aimed at the discovery and characterization of novel bioactive natural products.

## 1 Introduction

Edelweiss [*Leontopodium nivale* ssp. *alpinum* (Cass.) Greuter, syn.] is a rare, herbaceous traditional medicinal plant belonging to the Asteraceae family that can be found in Central Europe. All parts of the plant were widely applied in human and veterinary medicines before the species was overexploited and became endangered. Extracts from Edelweiss were used to treat different diseases, including gastrointestinal (dysentery and diarrhea), pulmonary (bronchitis), and heart conditions in humans, most likely because of its anti-inflammatory and antimicrobial properties mediated by bisabolane-type sesquiterpenes (Dobner et al., 2003). Recent research revealed that medicinal plants harbor endophytes, microorganisms living within plant tissues without causing disease symptoms to their host, some of which produce either precursor molecules or the actual bioactive compounds previously attributed to their host plants (Golinska et al., 2015). Host plants provide a nutrient-rich environment to their microbial inhabitants and shelter them from adverse environmental factors (Oukala et al., 2021). This spatially limited habitat leads to high selective pressure and promotes the establishment of a competitive endophytic community. Interactions between the community members likely involve secondary metabolites as growth-inhibiting and/or signaling molecules produced by some of its members in response to the excessive proliferation of the others (Scherlach and Hertweck, 2020). Thus, the bioprospecting of microorganisms inhabiting medicinal plants is a promising way to discover novel natural products in this yet underexplored environmental niche (Zotchev, 2024). These natural products may serve as lead molecules for the development of drugs to treat various diseases, including infections and cancer (Atanasov et al., 2021).

Among other genera of the phylum Actinobacteria, the genus *Streptomyces* produces a large number of compounds, some of which are of major importance for biotechnology, agriculture, and especially medicine, representing about two-thirds of all naturally derived antibiotics in current clinical use (Bérdy, 2005). The diversity of chemical structures of secondary metabolites from the genus *Streptomyces* encompasses non-ribosomal peptides, polyketides, ribosomally synthesized and post-translationally modified peptides, terpenes, indoles, quinones, and many other compounds (Hwang et al., 2014; Lacey and Rutledge, 2022). This chemical diversity also features various biological activities. Compounds isolated from *Streptomyces* species that are in clinical use include antifungals (e.g., amphotericin B and nystatin), anthelmintics (e.g., avermectin), antitumorals (e.g., doxorubicin), and immunosuppressives (e.g., rapamycin), as well as many antibacterial antibiotics (e.g., daptomycin, chloramphenicol, and tetracyclines) (Procópio et al., 2012). *Streptomyces* species are frequently isolated from various environmental sources, including medicinal plants (Golinska et al., 2015; Wu et al., 2021). The isolation of novel species and/or novel bioactive molecules from endophytic streptomycetes derived from medicinal plants has shown great potential (Zotchev, 2024). European medicinal plants represent a rather untapped source of streptomycetes and compounds isolated from them. For example, several diketopiperazines and the antifungal compounds cycloheximide and actiphenol were shown to be produced by the endophytic *Streptomyces* species isolated from *Arnica montana* (Wardecki et al., 2015). Other examples of European medicinal plants, where *Streptomyces* species could be isolated include *Arctium lappa, Convallaria majalis, Fragaria vesca, Melilotus officinalis, Rubus idaeus, Tanacetum vulgare, Taraxacum officinale, Trifolium pratense*, and *Urtica dioica*, a majority among them listed in the Pharmacopoeia Europaea 2022 (Golinska et al., 2015). However, secondary metabolites that these endophytic *Streptomyces* species from European medicinal plants can produce are still understudied. New approaches based on genome sequencing of these isolates combined with state-of-the-art analytical chemistry can help to solve this problem.

The biosynthesis of secondary metabolites in bacteria, including *Streptomyces*, is governed by the so-called biosynthetic gene clusters (BGCs), which encode enzymes that act in a coordinated fashion to assemble and modify molecular scaffolds (Medema et al., 2015). Many of these BGCs are typically transcriptionally “silent,” meaning they are not or only poorly expressed in standard laboratory conditions, resulting in no or only scarce secondary metabolite production in quantities insufficient for isolation and further testing. It has been shown that the genetic potential of *Streptomyces* and thus, the number of secondary metabolites they can produce, is far greater than could be identified upon conventional cultivation (Hopwood, 2019). The bioinformatics tool antiSMASH (Blin et al., 2023), among others, can detect such BGCs within genome sequences and, when combined with advanced metabolomics, help to identify and connect the secondary metabolites to their cognate BGCs.

In this study, nine bacterial strains belonging to the genus *Streptomyces* were isolated from Edelweiss and explored for their genetic potential to produce secondary metabolites. This was followed by metabolomics studies, which revealed the production of several known but also potentially new compounds, some of which could be linked to their cognate BGCs.

## 2 Materials and methods

### 2.1 Bacterial endophyte isolation and identification

The sterilization and maceration of the Edelweiss plant material are described in Oberhofer et al. (2021). For the isolation of endophytes, the obtained rhizome macerates were resuspended in water and 100 μl of each serial dilution was spread on the surface of six different nutrient media. Potato dextrose agar (PDA) and synthetic nutrient-poor agar (SNA) were prepared without antifungals, while glucose asparagine (GAC), humic acid vitamin agar (HVA), international *Streptomyces* project 2 agar (ISP2), and King’s B agar were supplemented with 20 μg/ml nystatin and 20 μg/ml cycloheximide to prevent fungal growth (see Supplementary material for media composition). All plates with plant macerates in different dilutions were incubated at 22°C. Petri dishes were monitored for 2 months, and selected colonies were subcultured and purified on tryptic soy agar (TSA) medium at room temperature. Resulting pure cultures were used to inoculate 3 ml of tryptic soy broth (TSB, Oxoid, UK) and incubated at 28°C, 200 rpm on a rotary shaker to obtain liquid cultures. A total of 800 μl of liquid culture was diluted 1:1 with 100% glycerol in cryo tubes and stored at −80°C. Sporulating isolates showing a three-dimensional shape morphologically resembling actinomycete bacteria were grown on soy flour mannitol agar (SFM) and Czapek’s agar (CP-6) (see Supplementary Table 1). Spore suspensions of each strain were prepared for storage at −80°C in 20% glycerol and grown in TSB and twofold yeast extract tryptone (2xYT) liquid medium to provide biomass for genomic DNA. The DNA isolation was done using Wizard^®^ Genomic DNA Purification Kit (Promega Corporation, Madison, WI, United States) following the manufacturers’ instructions. Quality and concentration of the purified genomic DNA were evaluated by gel electrophoresis in a 0.8% agarose gel supplemented with GelRed DNA stain (Biotium, Inc., Fremont, CA, United States).

PCR amplification of the 16S rDNA gene fragments was performed using the purified genomic DNA of each strain as a template with the standard forward (27F: 5′-AGAGTTTGATCMTGGCTCAG-3′) and reverse (1492R: 5′-TACGGY TACCTTGTTACGACTT-3′) primers as described in Hongoh et al. (2003). The amplified DNA fragments were verified with gel electrophoresis and products of expected length were purified using DNA Clean & Concentrator™-5 (Zymo Research, Irvine, CA, USA) or Zymoclean™ Gel DNA Recovery Kit (Zymo Research, Irvine, CA, USA) and sent to Eurofins Genomics (Eurofins Genomics AT GmbH, Vienna, Austria) for sequencing. The obtained sequences were queried using nucleotide BLAST, the EzBioCloud database (Yoon et al., 2017), and the Ribosomal Database Project (RDP) classifier (Maidak et al., 1996) to taxonomically classify the bacterial endophyte isolates at the level of genus.

### 2.2 Genomic DNA isolation and whole genome sequencing

DNA was extracted from cell pellets using the PowerSoil Pro DNA extraction kit (Qiagen) following the manufacturer’s protocol. DNA quality and quantity were assessed by fragment analysis using Genomic DNA tapes on a TapeStation 4100 (Agilent) and fluorometric DNA concentration was determined with a BR-dsDNA kit on a Qubit4 fluorometer (Thermo Fisher). Whole Genome Sequencing was performed on the Illumina and Oxford Nanopore Technologies (ONT) platforms at the Joint Microbiome Facility of the Medical University of Vienna and the University of Vienna (JMF) under project ID JMF-2104-14. DNA was prepared for Nanopore sequencing with the rapid barcoding sequencing kit (SQK-RBK110-96; ONT) following the manufacturer’s protocol. Barcoded DNA samples were sequenced with a Promethion P24 (ONT) on an R9.4.1 flow cell (FLO-PRO002; ONT) using Minknow (v. 21.10.8; ONT). For Illumina sequencing, DNA libraries were prepared from aliquots of the same extracts with the NEBNext Ultra II FS DNA library prep kit (New England Biolabs) and sequenced on the Illumina MiSeq platform (v3 chemistry, 2 × 300 cycles). Nanopore reads were base called using Guppy (v. 5.0.17) in super accuracy mode, while Illumina reads were quality trimmed using cutadapt (v. 3.1) (Martin, 2011) before further processing. The Nanopore reads were assembled using flye (v. 2.9-b1768) (Kolmogorov et al., 2019) with “–nano-hq,” polished twice times with minimap2 (v. 2.17) (Li, 2018) and racon (v. 1.4.3) (Vaser et al., 2017), twice with medaka (v. 1.4.4^1^), and finally with the Illumina data using minimap2 (v. 2.17) (Li, 2018) and racon (v. 1.4.3) (Vaser et al., 2017). Reads were mapped to the assemblies using minimap2 (v. 2.17) (Li, 2018) and read mappings were converted using samtools (v. 1.12) (Li et al., 2009) and read coverage was calculated using metabat2 (v. 2.15) (Kang et al., 2019). Genome quality was assessed using QUAST (v. 5.0.2) (Gurevich et al., 2013), CheckM (v. 1.1.1) (Parks et al., 2015), and genomes were classified using GTDB-Tk (v. 1.5.0) (Chaumeil et al., 2019).

All raw sequencing data and genomes generated in this study have been deposited to NCBI under the BioProject ID PRJNA1024677.

### 2.3 Analyses of genomes for secondary metabolites production potential

To compare the assembled and annotated genomes of the selected *Streptomyces* isolates, a nearest-neighbor analysis was performed using the TYGS database (Type Strain Genome Server, Leibniz Institute, DSMZ-German Collection of Microorganisms and Cell Cultures GmbH, Braunschweig, Germany) to build a genome-based phylogenetic tree (Meier-Kolthoff and Göker, 2019). The genome files were uploaded to the server to receive a list of matches. The accession number of the best match to each strain was entered into the server together with the *Streptomyces* genome sequences again to gain the final phylogenetic tree. The endophytic *Streptomyces* species were further mined for potential secondary metabolite BGCs using the online bioinformatics tool antiSMASH 7.0 (Blin et al., 2023). The identified BGCs were manually analyzed for potential novelty and uniqueness, mainly focusing on BGCs encoding non-ribosomal peptide synthases (NRPS), polyketide synthases (PKS), ribosomally synthesized and post-translationally modified peptides (RiPPs), and hybrid gene clusters.

### 2.4 Secondary metabolite production by endophytic *Streptomyces* spp.

The strains were streaked onto SFM or CP-6 agar, depending on the isolate (see Figure 2) and incubated at 28°C. Typically after 96–168 h, the spores were collected and stored in 2 ml 20% glycerol at −80°C. A total of 100 μl of spore suspension was used to inoculate 10 ml TSB and 2xYT medium (see Supplementary Table 3 for media composition) to prepare a seeding culture, grown at 28°C at 200 rpm on a rotary shaker. After sufficient growth, typically after 48–96 h, 2.5 ml of the seeding culture was used to inoculate 50 ml GYM, SM17, or SG liquid fermentation medium (see Supplementary Table 3 for media composition) in 250 ml baffled flasks, which were then incubated at 28°C, 200 rpm. After 8 days of fermentation, the cultures were freeze-dried and extracted with 50 ml methanol for 2 h at 150 rpm at room temperature. The crude extracts were transferred to 50 ml Falcon™ tubes and centrifuged at 4,000 rpm for 20 min at room temperature. The extracts’ supernatants were decanted to a 100 ml round-shaped flask, and the solvent was removed under reduced pressure using a rotary evaporator. The dried samples were re-suspended in 3.5 ml methanol and stored at −20°C. Analytical HPLC analysis was performed with a Shimadzu system consisting of a CBM-20A system controller, an LC-20A solvent delivery pump, a DGU-20A5 degasser, a SIL-20A autosampler, a CTO-20AC column oven, an SPD-M20A diode array detector, and an evaporative light scattering detector LT-II (ELSD LT-II). The column oven temperature was set to 25°C. The autosampler was set to inject 10 μl of extract and for separation, a Phenomenex Luna^®^ C18 column as stationary phase (C18, 4.6 mm × 150 mm, 5 μm) was used. Two solvents (solvent A, 0.1% aqueous solution of formic acid; solvent B, acetonitrile) were used as mobile phase with a flow rate of 1 ml/min and a gradient over 65 min. During gradient elution, solvent B was increased from 5% to 95% over 45 min and held for 10 min. After 55 min, the column was washed and re-equilibrated for 10 min with 5% of solvent B.

### 2.5 Antimicrobial activity testing

The antimicrobial activity of extracts and fractions was evaluated using several test organisms including *Bacillus subtilis* DSMZ 10, *Enterococcus mundtii* DSMZ 4840, *Kocuria rhizophila* DSMZ 348, *Micrococcus luteus* DSMZ 1790, *Staphylococcus carnosus* DSMZ 20501, *Escherichia coli* DH5α, *Pseudomonas putida* KT2440, and *Saccharomyces cerevisiae* BY4742 (Supplementary Table 13). A total of 50 μl of crude methanolic extracts, fractions, or pure compounds along with a positive (antibacterial bioassays: 100 μg apramycin, yeast: 20 μg cycloheximide) and negative controls (methanol as solvent control) were applied onto a 9 mm sterile paper disc (Whatman GE Healthcare Life Sciences, USA). The discs were dried under sterile conditions to remove the solvent before being placed onto specific growth media inoculated with 200 μl cell suspension of test organism in 20% glycerol. Bacterial tests were done on Lennox or TSA agar at 28°C or 37°C, depending on the bacterial strain, while yeast was incubated on yeast extract peptone dextrose (YPD) agar at 28°C overnight, and inhibition zones were measured the following day.

### 2.6 LC-MS analyses and secondary metabolite dereplication

LC-MS analyses of the culture extracts were performed on a Vanquish Horizon UHPLC system (Thermo Fisher Scientific) coupled to the ESI source of a timsTOF fleX mass spectrometer (Bruker Daltonics). Separation was carried out on an Acquity Premier HSS T3 column, 2.1 × 150 mm, 1.8 μm (Waters) using water and acetonitrile/water 9:1, both modified with 0.1% formic acid, as mobile phase A and B, respectively. The sample components were separated and eluted with a gradient starting with a linear increase from 0% to 20% B in 10 min, followed by a linear increase from 20% to 100% B in 25 min, and finally an isocratic column cleaning (3 min at 100% B) and re-equilibration step (5 min at 0% B). The flow rate was 0.5 ml/min, and the column oven temperature was set to 40°C. High-resolution ESI-MS and MS/MS spectra were recorded in positive ion mode in the range of *m/z* 100-2500. Collision-induced dissociation (CID) mass spectra of the five most intense precursor ions in each MS^1^ spectrum were obtained in automated data-dependent acquisition mode using nitrogen as collision gas. The sum formulae of the detected ions were determined using Bruker Compass DataAnalysis 5.3 based on the mass accuracy (Δm/z ≤ 5 ppm) and isotopic pattern matching (SmartFormula algorithm). Compounds represented in GNPS were identified by a MOLECULAR-LIBRARYSEARCH-V2 (release_28) workflow (Wang et al., 2016), whereby in the case of the very common hydroxamate siderophores only the main congeners present in the extracts are listed in Supplementary Table 15. Next, MZmine 3 was used for aligning and comparing the LC-MS data of all nine strains separately for each medium (SM17, SG, and GYM) to find strain-specific secondary metabolites (Schmid et al., 2023). Additional known secondary metabolites were then identified using The Natural Products Atlas (van Santen et al., 2022) and CAS SciFinder (American Chemical Society). All identifications were manually validated by comparison of the obtained MS/MS and DAD spectra with available referenced data and/or thorough spectra interpretation.

## 3 Results and discussion

### 3.1 Genome analysis

A total of 609 bacterial isolates were obtained from three tissues (leaves, roots, and rhizomes) of five different *L. nivale* ssp. *alpinum* plants (LN1–LN5). At least nine of these isolates showed an actinomycete-like morphology on the isolation plates and were identified as members of the genus *Streptomyces* by 16S rRNA gene-based taxonomy. All *Streptomyces* strains in this study originated from the rhizomes of four different plant specimens. Strains LN245, LN549, LN699, and LN785 were derived from the plant specimen LN1. The isolates LN499, LN500, and LN590 were isolated from the rhizome tissue of the host plant LN2. Isolates LN325 and LN704 originated from the plants LN5 and LN3, respectively.

**FIGURE 1:**
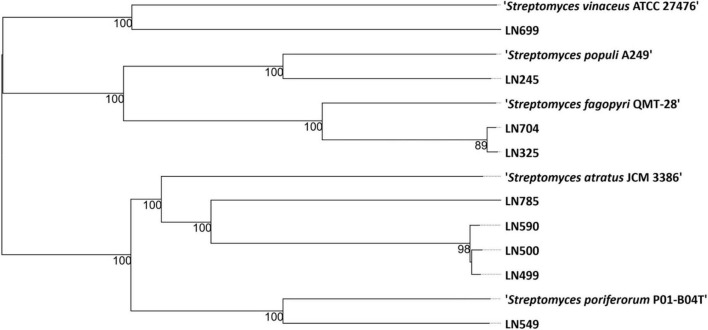
Phylogenetic tree based on the genome sequences from nine *Streptomyces* spp. isolated from Edelweiss and the closest related type strains (nearest neighbors) in the TYGS database (Type Strain Genome Server, Leibniz Institute, DSMZ-German Collection of Microorganisms and Cell Cultures GmbH, Braunschweig, Germany) (Meier-Kolthoff and Göker, 2019).

The genomes of the endophytic *Streptomyces* isolates were sequenced and used to generate a genome-based phylogenetic tree (Figure 1) and to assess their secondary metabolite production potential (see section “2 Materials and methods”). According to the phylogenetic position of the Edelweiss isolates in the genome-based tree, they may represent new species of the genus *Streptomyces*. The closest type strain related to *Streptomyces* sp. LN245 was *Streptomyces populi* A249, an endophyte isolated from the stem of *Populus adenopoda* collected at Mount Qingcheng in southwest China (Wang et al., 2018). The closest match for both *Streptomyces* spp. LN325 and LN704 was *Streptomyces fagopyri* QMT-28 isolated from the rhizosphere of *Fagopyrum dibotrys* collected in Shuangfeng, Hunan Province, China (Guo et al., 2020). For LN499, LN500, LN590, and LN785, the nearest neighbor from the TYGS database was found to be *Streptomyces atratus* JCM 3386, which was isolated from soil in Shimoneda in Japan (Shibata et al., 1962). Besides being closely related based on the genome-based phylogeny, the LN499, LN500, and LN590 isolates also exhibited similar morphology on solid media. *S. atratus* is known to produce several antibacterial and cytotoxic compounds, including ilamycins (Ma et al., 2017), rufomycins (Shibata et al., 1962), atramycins (Fujioa et al., 1991), and hydrazidomycins (Ueberschaar et al., 2011). For LN549, marine sponge-derived *Streptomyces poriferorum* P01-B04T, isolated from *Geodia barretti* from the Trondheim fjord of Norway, was the only closely related type strain in the TYGS database. The latter strain has been shown to have antimicrobial activity against Gram-positive bacteria (Sandoval-Powers et al., 2021). The closest type strain related to *Streptomyces* sp. LN699 from the TYGS database was *Streptomyces vinaceus* ATCC 27476, a soil-derived isolate known to produce arginine-derived polyketides (arginoketides), which mediate cross-kingdom microbial interactions with fungi of the genera *Aspergillus* and *Penicillium* and trigger the production of secondary metabolites in the latter (Krespach et al., 2023).

**FIGURE 2:**
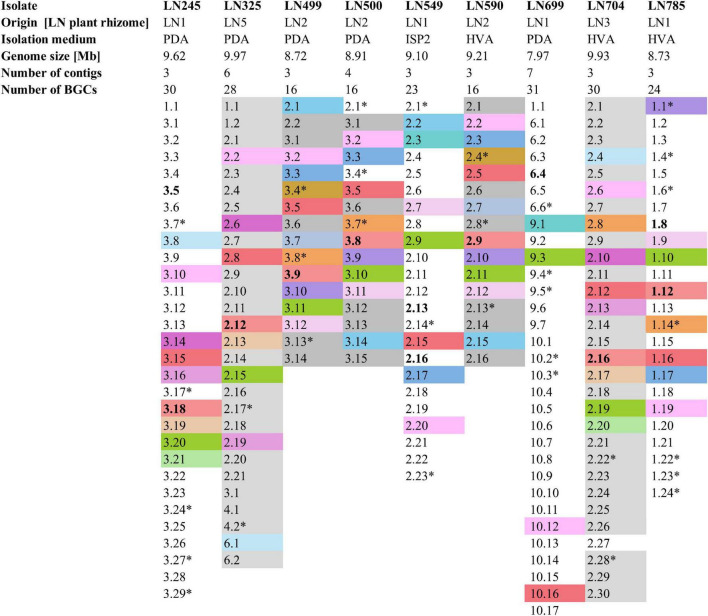
Summary of the Edelweiss isolates’ origin, isolation medium, genome size, number of contigs, and BGCs. BGCs shaded the same color represent BGCs shared between two or more of these isolates. Non-shaded BGCs are unique for each isolate. BGCs presumed to be unique are marked by a star sign (*). BGCs putatively specifying the biosynthesis of the identified secondary metabolites by high-resolution LC-MS are marked in bold. Detailed antiSMASH-based BGC analyses are given in Supplementary Tables 4–12. PDA, potato dextrose agar; ISP2, international *Streptomyces* project 2 agar medium; HVA, humic acid vitamin agar.

**FIGURE 3:**
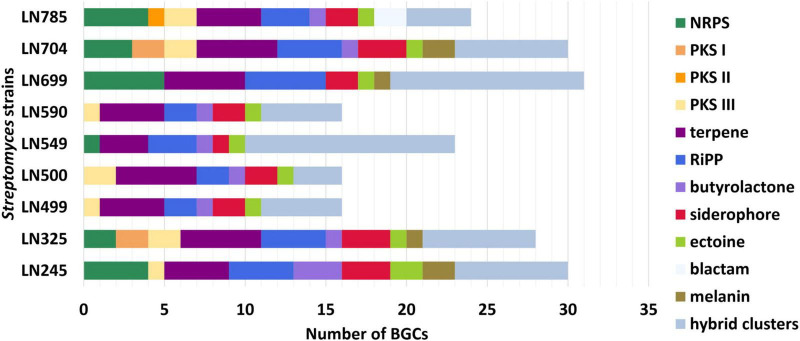
Numbers and types of biosynthetic gene clusters (BGCs) identified in *Streptomyces* endophytes. NRPS, nonribosomal peptide synthetases; PKS, polyketide synthases; RiPP, ribosomally synthesized and post-translationally modified peptides.

The lists of BGCs detected by antiSMASH and representing the biosynthetic potential of the *Streptomyces* isolates are given in Supplementary Tables 4–12. A total of 214 BGCs were identified in nine genomes, ranging from 16 (LN499, LN500, and LN590) to 31 (LN699) per *Streptomyces* isolate as illustrated in Figure 2, and their distribution per isolate are shown in Figure 3. Based on manual analyses, 35 of these clusters were tentatively marked as unique, meaning that the BGC gene composition did not match any BGCs in available public databases such as MiBIG (Terlouw et al., 2023). Analyses of the BGCs from Edelweiss-derived strains revealed that LN325 (Supplementary Table 5) shares its 28 BGCs with LN704 (Supplementary Table 11), which contains additional two clusters, BGC 2.20, and BGC 2.27. Analysis of the antiSMASH data for LN325 and LN704 by ClusterBlast function, which shows the presence of genes from certain BGCs in other bacteria, revealed that next to *S. fagopyri* QMT-28, LN325 and LN704 share several BGCs with *Streptomyces* RPA4-2, an isolate from French forest soil (Nicault et al., 2020). LN499 (Supplementary Table 6), LN500 (Supplementary Table 6), and LN590 (Supplementary Table 7) harbor and share the smallest number of BGCs (16) among the studied isolates, with LN499 and LN590 having seemingly identical BGCs. The latter isolates differ from LN500 in just two BGCs. BGC 3.7 from LN499 and BGC 2.7 from LN590 represent the same PKSI-NRPS hybrid cluster which is not present in LN500. On the other hand, PKSIII BGC 2.1 from LN500 is absent in both LN499 and LN590. The second difference is the presence of PKSII BGC 3.4 in LN500, which is “replaced” with arylpolyene-specifying BGC 3.4 and BGC 2.4 in LN499 and LN590. Notably, exactly those clusters that were found to be different were marked as unique when using the antiSMASH ClusterBlast and KnownClusterBlast functions (Supplementary Tables 6, 7, 9).

### 3.2 Secondary metabolites produced by the Edelweiss-derived *Streptomyces* spp.

#### 3.2.1 Antimicrobial activity testing of the culture extracts

The methanolic extracts from *Streptomyces* spp. cultures grown in different media were tested for antimicrobial activity against bacteria and yeast (see section “2 Materials and methods”). Among all the extracts, only the ones from *Streptomyces* sp. LN549 showed inhibition of Gram-positive bacteria *M. luteus, B. subtilis, K. rhizophila*, and *S. carnosus*. The extract from *Streptomyces* sp. LN699 culture grown in MYM medium showed activity against *S. cerevisiae* and *B. subtilis.* None of the other extracts exhibited antimicrobial activities in the conditions tested. Full results of the antimicrobial activity testing of the Edelweiss-derived *Streptomyces* isolates are presented in Supplementary Table 14.

#### 3.2.2. Secondary metabolomes of the *Streptomyces* isolates and their links to BGCs

All the extracts generated for the *Streptomyces* isolates were analyzed using high-resolution LC-MS (see section “2 Materials and methods”). After the analyses, GNPS (Wang et al., 2016), The Natural Products Atlas (van Santen et al., 2022) and CAS SciFinder (American Chemical Society) were used to identify known compounds based on the predicted sum formula and plausible match of MS/MS and DAD data. The LC-MS results are listed in Supplementary Table 15. To investigate the possible biosynthetic origins of the identified secondary metabolites from the extracts of each Edelweiss isolate, the BGCs detected by antiSMASH were analyzed with respect to their ability to specify the biosynthesis of identified secondary metabolites. This was done via extensive literature search as well as existing knowledge on the biosynthesis of natural products.

In extracts of LN245, several members of the ferrioxamine family, including the well-known hydroxamate siderophore desferrioxamine B were identified (Barona-Gómez et al., 2004). Indeed, BGC 3.18 (Supplementary Table 4) contains genes involved in desferrioxamine B and E biosynthesis as detected by antiSMASH. Desferrioxamine B was first isolated from *Streptomyces pilosus* in 1960 (Bickel et al., 1960) and is being used in medicine as a metal chelator under the brand name Desferal to remove excess iron or aluminum from the blood (Shaer et al., 2020). Siderophore production of endophytes promotes competitive advantages in colonizing plant tissues and suppresses other microorganisms from the same ecological niche via the sequestration of iron, an essential co-factor of many enzymes (Loaces et al., 2011). On the other hand, plants profit from the growth-promoting functions of the metal- and phosphor-scavenging siderophores produced by endophytes (Cui et al., 2022; Timofeeva et al., 2022).

Putatively new sulfur-containing natural products, congeners with the sum formulae C_19_H_34_N_2_O_7_S, C_20_H_36_N_2_O_7_S, and C_21_H_38_N_2_O_7_S, could be identified in the extracts from LN245. Since no sulfotransferase-encoding gene could be identified within the BGCs of LN245 by antiSMASH, it was impossible to link these putatively new compounds to a particular BGC.

In the extracts of strain LN325, desferrioxamine B, and two tentatively new desferrioxamine-like siderophores could be detected by LC-MS. They might originate from BGC 2.12 (Supplementary Table 5), which contains genes involved in desferrioxamine B and E biosynthesis. Interestingly, the putatively new siderophores appear to contain a carboxyl group instead of an amino group at the C terminus (data not shown).

*Streptomyces* sp. LN499 extracts analyses revealed the presence of three known hydroxamate siderophores, desmethylenylnocardamine, dehydroxynocardamine, and desferrioxamine E as main secondary metabolites. The BGC that codes for the enzymes needed for their synthesis is most likely BGC 3.9, specifying the biosynthesis of desferrioxamine B and E production.

As for LN499, the extracts of the closely related strain LN500 mainly contained siderophores from the ferrioxamine family, namely desferrioxamine B and E, desmethylenylnocardamine, and legonoxamine A, whose biosynthesis is likely to be specified by BGC 3.8 (Supplementary Table 7). Additionally, a compound with the assigned sum formula C_15_H_18_N_2_O_2_S could be detected as [M + H]^+^ ion at *m/z* 291.1160 in the LN499 extract. We could not connect this compound to any BGC in the LN499 genome, suggesting that it might represent a degradation product originating from a methionine- or/and cysteine-containing peptide.

**FIGURE 4:**
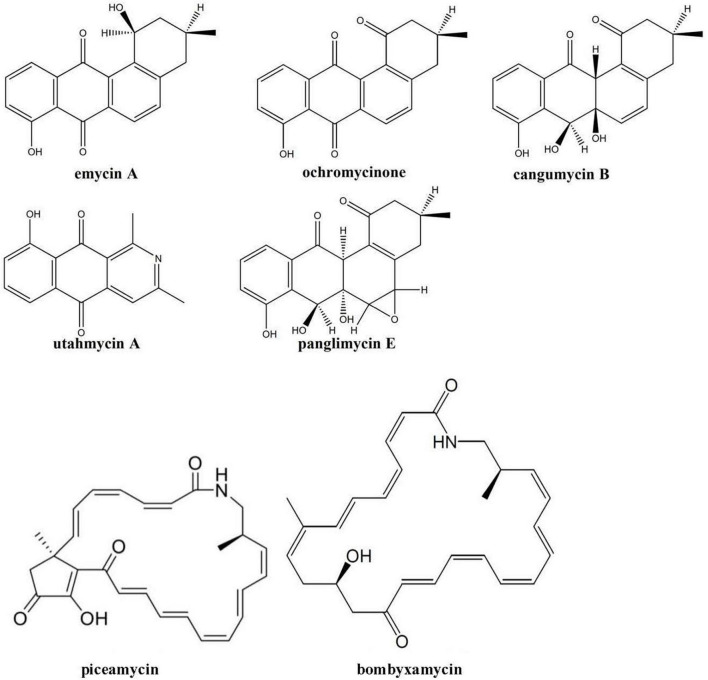
Secondary metabolites tentatively identified using high-resolution LC-MS in the extracts of *Streptomyces* sp. LN549.

In contrast to the secondary metabolomes of other Edelweiss-derived *Streptomyces* isolates, the extracts from *Streptomyces* sp. LN549 cultures did not contain ferrioxamine siderophores. At the same time, several angucyclinones including known compounds and many of their isomers were detected. In particular, panglimycin E (Fotso et al., 2008), cangumycin (Wang et al., 2019), ochromycinone (Zhang et al., 2021), and emycin A (Larsen et al., 1996), could be putatively identified (Figure 4). Ochromycinone is known to inhibit the growth of *B. subtilis* and *Pseudomonas aeruginosa*, which might explain the bioactivity of LN549 extracts against Gram-positive bacteria, while activity against Gram-negative *E. coli* and *P. putida* used in our antimicrobial tests was not observed. As shown by Fotso et al. (2008), such angucycline polyketides and a large number of congeners often appear in extracts together, since post-PKS tailoring reactions can generate a huge structural diversity. The biosynthetic origin of the identified angucyclinones could be tentatively connected to BGC 2.16 (Supplementary Table 8), a hybrid PKSII-indole cluster, which is related to a BGC from *Streptomyces* sp. CB02414 responsible for the biosynthesis of several rubiginones and ochromycinone. The latter is a precursor of rubiginone and is modified by an O-methyltransferase to gain rubiginone B_2_ (Zhang et al., 2021). Utahmycin A, a 2-azaanthraquinone compound, putatively identified by LC-MS, might have its origin in BGC 2.16 as well. Comparing the structures of utahmycin A to the detected angucyclinones, they share the anthraquinone skeleton, but utahmycin A does not contain a fourth ring and a difference is the incorporation of nitrogen into the molecule (Figure 4). Utahmycin A was first described upon heterologous expression of an environmental DNA clone in *Streptomyces albus* J1074 and was predicted to be derived from the erdacin biosynthetic pathway (Bauer et al., 2010). *Streptomyces* sp. AK 671 has been reported to produce utahmycin A, which might be formed from the known chrysophanol precursor genoketide A1 just by nitrogen incorporation and oxidation to the azaanthraquinone. However, the biosynthetic origin of the nitrogen remains unclear for both the fungal-derived 2-azaanthraquinones and the utahmycins (Jetter et al., 2013). Other natural products identified in the extracts of LN549 were the known isoflavonoids daidzein-rhamnoside as well as two putatively new glycosylation products of daidzein (C_27_H_30_O_11_) and genistein (C_21_H_20_O_6_), presumably derived from the soy flour as a component of SM17. *Streptomyces* has been shown to utilize isoflavone glycosides for growth promotion by microbial glycosylation of isoflavones (Wewengkang et al., 2020). Additionally, extracts of LN549 contained two known compounds putatively identified as the macrocyclic lactams bombyxamycin A and piceamycin (Figure 4). The former was first identified in *Streptomyces* sp. SD53 isolated from the gut of the silkworm *Bombyx mori* (Shin et al., 2019) and the latter in *Streptomyces* sp. GB4-2 isolated from the mycorrhizosphere of *Picea abies* (Schulz et al., 2009). Numerous congeners of these compounds have also been identified. Piceamycin and bombyxamycin were shown to exhibit cytotoxic and antimicrobial activities and could thus be responsible for the activity against Gram-positive bacteria observed in disc diffusion assays with the LN549 extracts. An upscaled fermentation followed by a bioactivity-guided fractionation using semi-preparative HPLC was performed to purify the antimicrobial secondary metabolite from the LN549 extract. To confirm the identity of the presumed antimicrobial compound, the active fraction was subjected to analytical HPLC-DAD analysis. A corresponding peak in the chromatogram at 32.93 min with two absorption maxima (295 and 410 nm) indicated the presence of piceamycin, whereas bombyxamycin A or congeners thereof were not detected. In the genome of LN549, BGC 2.13 (Supplementary Table 8), a PKSI-PKSII hybrid cluster, is most likely responsible for piceamycin biosynthesis. The results from antiSMASH version 7.0.1 indicated the presence of a few genes for bombyxamycin biosynthesis in BGC 2.13. Indeed, both macrolactams originate from the same biosynthetic pathway as shown by Grubbs et al. for *Streptomyces* sp. AmelAP-1, where the biosynthesis in addition to the structure elucidation and the antimicrobial activity has been reported (Grubbs et al., 2021).

In the extracts from cultures of LN590, the same hydroxamate siderophores (desferrioxamine B, and E, desmethylenylnocardamine, and legonoxamine A) were identified as in extracts of the most closely related strain LN500. BGC 2.6 (Supplementary Table 9) encoding for desferrioxamine B and E biosynthesis, as detected by antiSMASH, is most likely responsible for the siderophores’ production. Interestingly, the tentatively new compound detected in LN500 extracts (C_15_H_18_N_2_O_2_S), could be identified in extracts of LN590 as well.

**FIGURE 5:**
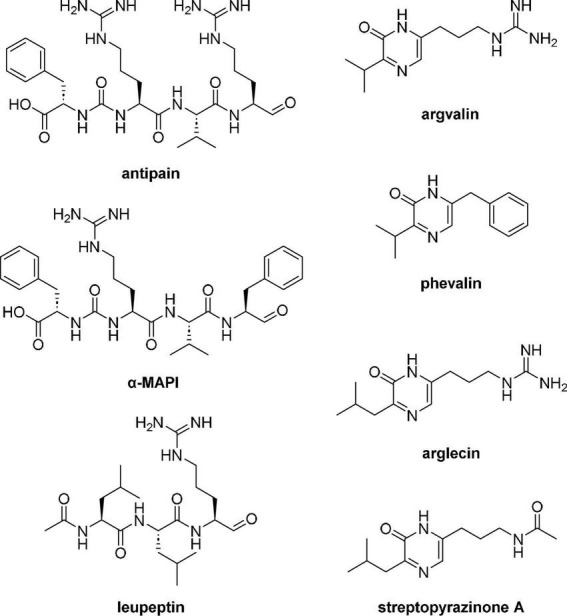
Secondary metabolites tentatively identified using high-resolution LC-MS in the extracts of *Streptomyces* sp. LN699.

As for LN549, extracts from *Streptomyces* sp. LN699 did not contain hydroxamate siderophores but showed antimicrobial activity against *S. cerevisiae* and *B. subtilis*. Notably, among the Edelweiss isolates these two strains were the only ones lacking a desferrioxamine D/E BGC in their genomes. These two strains were shown to produce secondary metabolites of potential pharmaceutical relevance. A large number of known and unknown non-ribosomally synthesized peptides, including antipain-like protease inhibitors and pyrazinone derivatives were putatively identified. The antipain-like small peptides identified include antipain (Suda et al., 1972), reduced antipain, leupeptin (Aoyagi et al., 1969), reduced leupeptin, leupeptin Pr-LL (Kondo et al., 1969), metabolite KF77-AG6 (Fujimoto et al., 1974), MAPI (microbial alkaline protease inhibitor, Watanabe et al., 1979), Mer-N5075-A (reduced MAPI, Kaneto et al., 1993), oxidized MAPI, and unknown congeners. The vast number of congeners seems to appear due to the spontaneous reaction of the aldehyde moiety in antipain, leupeptin, and MAPI (Figure 5). These compounds are known serin and cysteine protease inhibitors and have been reported to possess antiviral activities, for example, antipain against HCoV-229E and poliovirus 2A, and leupeptin against HCoV-229E and influenza virus (Sun et al., 2022). Since these compounds are produced by NRPSs, the corresponding BGC in LN699 was identified as the hybrid BGC 6.4 (Supplementary Table 10) that exhibits 100% gene sequence identity with antipain BGC in the MIBiG database. Phylogenetic studies on the genetic basis of the identified aldehyde-containing, peptidic protease inhibitors and heterologous expression of the deimino-antipain BGC have shown that they likely share a common biosynthetic origin (Maxson et al., 2016). Notably, leupeptin has been shown to derive from a non-NRPS pathway, which uses separate AMP-dependent ligases to form amide bonds and a reductase to form the aldehyde in *Xenorhabdus bovienii* SS-2004 (Li et al., 2020). The obvious substrate promiscuity of the NRPS in the antipain biosynthesis and the reactive aldehyde group in the final molecule likely leads to the expansive production of the identified congeners. In addition to the NRPs mentioned above, several other NRPS-derived pyrazinone derivatives (Figure 5), including phevalin (Alvarez et al., 1995), argvalin (Tatsuta et al., 1973), arglecin (Tatsuta et al., 1971), and streptopyrazinone A or D (Chen et al., 2018) were putatively identified. Considering that the main pyrazinones are composed of the same amino acids as the protease inhibitors, we assume that they are produced by spontaneous cyclization of the terminal aldehyde (Arg or Phe) with the nitrogen of the adjacent amino acid (Val or Leu) followed by oxidation, either as side product during biosynthesis or as degradation product of the final reactive compounds in solution. Streptopyrazinone A could then be biosynthesized from arglecin by arginase-activity followed by acetylation of the resulting ornithine side-chain. Streptopyrazinones have been shown to inhibit fungal and bacterial growth (Chen et al., 2018), and thus might be responsible for the observed antimicrobial activity of LN699 extracts.

Another tentatively identified compound in the LN699 extract was aureonucleomycin, a nucleoside antibiotic produced by *Streptomyces aureus* var. *suzhoueusis* (Pan et al., 2019). The presence of this compound could also be responsible for the antimicrobial activity of LN699 extracts. However, the corresponding BGC reported by Pan et al. (2019) could not be detected in the genome of LN699, leaving the identity of the tentatively identified compound in doubt.

In the extracts from LN704, the main compounds identified were again siderophores including several isomers of desferrioxamine B and E, dehydroxynocardamine, and two unknown hydroxamate siderophore congeners. The two unknown congeners are, according to their accurate masses, the same compounds as identified in the extracts of LN325, which is phylogenetically closely related to LN704 (see Figure 1). The responsible BGC for the biosynthesis of several members of the ferrioxamine family identified in LN704 extracts was found to be BGC 2.16 (Supplementary Table 11).

Desferrioxamine B and E, dehydroxynocardamine, and legonoxamine A were also detected in the extracts of LN785. BGC 1.12 (Supplementary Table 12) is most likely to govern their biosynthesis since genes of this cluster show a similarity to genes involved in desferrioxamine B and E BGC from *Streptomyces griseus* ssp. *griseus* NBRC 13350. Other putatively identified compounds in LN785 extracts were WS-5995 C and a possible congener, known polyketide antibiotics with a naphthoquinone skeleton (Ikushima et al., 1980) most likely specified by the PKSII BGC 1.8 (Supplementary Table 13) described in Chen et al. (2022).

## 4 Conclusion

The genetic potential of the *Streptomyces* isolates from Edelweiss to produce diverse secondary metabolites has been shown by the antiSMASH analyses, detecting a total of 214 BGCs of which 35 were presumed to be unique. Several known but also potentially new secondary metabolites could be putatively identified in the extracts from these isolates using LC-MS-based dereplication. Among the identified compounds, some were shown to be bioactive (e.g., piceamycin), while others are known to have various bioactivities (e.g., angucyclinones) or are well-known drugs in clinical use (e.g., desferrioxamine B and several peptidic protease inhibitors). This work provides an example of how bioinformatics and metabolomics can be used to probe the biosynthetic capabilities of endophytic *Streptomyces* bacteria, which may aid drug discovery and help to better understand their ecological roles in the natural environment.

## Data availability statement

The original contributions presented in the study are publicly available. This data can be found at the National Center for Biotechnology Information (NCBI) using accession number PRJNA1024677.

## Author contributions

FM: Investigation, Methodology, Writing – original draft. MZ: Data curation, Investigation, Methodology, Writing – original draft, Writing – review & editing. RK: Investigation, Methodology, Writing – review & editing. MO: Investigation, Methodology, Writing – review & editing. SZ: Conceptualization, Project administration, Supervision, Writing – original draft, Writing – review & editing.

## Footnotes

1
https://github.com/nanoporetech/medaka
