# Supplementary material for: Genomes and secondary metabolomes of Streptomyces spp. isolated from Leontopodium nivale ssp. alpinum
Source: Front Microbiol. 2024 Jun 14;15:1408479. doi: 10.3389/fmicb.2024.1408479 (PMC11212599; doi:10.3389/fmicb.2024.1408479)
Supplement: Supplementary file 2 [file Data_Sheet_1.PDF]

Supplementary Material

Genomes and secondary metabolomes of *Streptomyces* spp. isolated from *Leontopodium nivale* ssp. *alpinum*

Fabian Malfent, Martina Oberhofer, Martin Zehl, Petra Pjevac, Joana Seneca Silva, Sergey B. Zotchev*

*** Correspondence:** sergey.zotchev@univie.ac.at

1 Supplementary Data

Table S1. Composition of nutrient media per one liter for isolation of microorganisms from Edelweiss.

Ingredients [g/L]	GAC agar	HV agar	ISP2 agar	Kings B agar	PDA agar	SNA Agar	TSA agar
Yeast extract	-	-	4.0	-	-	-	-
Malt extract	-	-	10.0	-	-	-	-
Dextrose	-	-	4.0	-	20.0	-	-
Glucose	2.0	-	-	-	-	0.2	-
Glycerol	-	-	-	10 ml	-	-	-
Casein peptone	-	-	-	-	-	-	15.0
Peptone	-	-	-	20.0	-	-	-
Soy peptone	-	-	-	-	-	-	5.0
Humic acid	-	1.0	-	-	-	-	-
Potato starch*	-	-	-	-	4.0	-	-
Sucrose	-	-	-	-	-	0.2	-
L-Asparagine	1.0	-	-	-	-	-	-
Humic acid	-	-	-	-	-	-	-
K_2HPO_4 x $3H_2O$	0.4	-	-	1.5	-	1.0	-
$CaCO_3$	-	0.02	-	-	-	-	-
NaCl	-	-	-	-	-	-	5.0
KCl	0.3	1.7	-	-	-	0.5	-
KNO_3	-	-	-	-	-	1.0	-
NaH_2PO_4	-	0,5	-	-	-	-	-
$MgSO_4$ x $7H_2O$	0.3	0.5	-	1.5	-	0.5	-
$FeSO_4$ x $7H_2O$	0.01	0.01	-	-	-	-	-
$CuSO_4$ x $5H_2O$	0.001	-	-	-	-	-	-
$ZnSO_4$ x $7H_2O$	0.001	-	-	-	-	-	-
$MnSO_4$ x $7H_2O$	0.001	-	-	-	-	-	-
B-vitamins**	-	+	-	-	-	-	-
Nystatin	+	+	+	+	-	-	-
Cycloheximide	+	-	-	-	-	-	-
Agar	20.0	18.0	20.0	15.0	12.0	15.0	15.0
pH	7.4	7.2	7.2	7.2	5.6	5.4	7.3

*approx. 200 g of infusion from potatoes
**0.25 mg.L^{-1} biotin and 0.5 mg.L^{-1} of thiamine HCl, riboflavin, niacin, pyridoxin HCl, inositol, Ca-pantothenate, p-aminobenzoic acid

Table S2. Composition of solid nutrient media per one liter for growth of *Streptomyces* strains.

Ingredients [g/L]	SFM agar	CP-6 agar	ISP2 agar
Corn Steep Liquor	-	10.0	-
Glucose	-	-	4.0
Malt extract	-	-	10.0
Soluble starch	-	10.0	-
Soy flour	20.0	-	-
Soy peptone	5.0	-	-
Yeast extract	-	-	4.0
$CaCO_3$	-	3.0	-
NaCl	-	3.0	-
$(NH_4)_2SO_4$	-	3.0	-
Agar	20.0	20.0	20.0
Tap water	+	+	-
ddH_2O	-	-	+
pH	7.0	7.2	7.2

Table S3. Composition of liquid nutrient media per one liter for growth and fermentation of *Streptomyces* strains. TSB and 2xYT were used for growth and preparation of seeding cultures, while the others were used for secondary metabolite production. All media, except SM17, were prepared using double-distilled water. The pH was adjusted with 1M sodium hydroxide or 1M hydrochloric acid.

Ingredients [g/L]	TSB	2xYT	SM17	GYM	MYM	SG
Casein peptone	15.0	-	-	-	-	-
Glucose	-	-	2.0	4.0	4.0	20.0
Glycerol	-	-	20.0	-	-	-
Malt extract	-	-	-	10.0	10.0	-
MOPS	-	-	-	-	1.9	-
Peptone	-	-	5.0	1.0	-	10.0
Soluble starch	-	-	2.0	-	-	-
Soy flour	-	-	5.0	-	-	-
Soy peptone	5.0	-	-	-	-	-
Tryptone	-	16.0	-	-	-	-
Yeast extract	-	10.0	5.0	4.0	4.0	-
$CaCO_3$	-	-	2.0	-	-	2.0
$CoCl_2$	-	-	-	-	-	0.001
NaCl	5.0	5.0	5.0	2.0	-	-
Tap water	-	-	+	-	-	-
pH	7.3	7.2	6.4	7.2	7.2	7.2

Tables S4 – S12. Tables with BGCs from nine Edelweiss *Streptomyces* isolates identified by antiSMASH 7.0. Potentially unique clusters (BGCs, which had a low sequence similarity with known BGCs, or if known than just a minor part of the genes from cluster encode for the putative product, and the low similarity to BGCs found in other bacteria) are in bold. **Table S4.**

***Streptomyces* sp. LN245**			
**Region**	**BGC type**	**Presence in another bacterium**	**Putative product**
1.1	lanthipeptide-class-iii	*Streptomyces* sp. FR-008 33%	SapB 100%
3.1	NRPS,T1PKS,other	*Streptomyces sp.* WAC 01529 51%	polyoxypeptin 64%
3.2	butyrolactone,T3PKS	*Streptomyces* sp. BK329 21%	lactonamycin 8%
3.3	butyrolactone	*Streptomyces pseudovenezuelae* strain DSM 40212 66%	neocarzinostatin 4%
3.4	butyrolactone	*Streptomyces mirabilis* strain OV308 66%	A-factor 100%
3.5	thioamitides	*Streptomyces* lydicus strain WYEC 108 10%	macrotermycins 7%
**3.6**	**NRPS,T1PKS,redox-cofactor**	***Micromonospora zamorensis* strain DSM 45600 15% (unique)**	**-**
**3.7**	**terpene**	***Streptomyces* sp. BK387 10% (unique)**	**-**
3.8	RiPP-like	Multiple *Streptomyces* spp. 100%	informatipeptin 42%
3.9	NRPS-like,NRPS,T1PKS, T2PKS	*Streptomyces* sp. 142MFCol3.1 51%	spore pigment 83%
3.10	terpene	Multiple *Streptomyces* spp. 100%	hopene 92%
3.11	siderophore	Multiple *Streptomyces* spp. 100%	paulomycin 7%
3.12	RiPP-like	*Streptomyces* sp. RPA4-2 50%	geosmin 100%
3.13	terpene	*Streptomyces* sp. RLB3-17 71%	albaflavenone 100%
3.14	siderophore	Multiple *Streptomyces* spp. 100%	thioviridamide 21%
3.15	terpene	*Streptomyces* sp. Tue6028 94%	desferrioxamin B / desferrioxamine E 83%
3.16	NRPS-like	*Streptomyces* sp. Go-475 31%	istamycin 5%
3.17	siderophore	Multiple *Streptomyces* spp. 100%	ectoine 100%
3.18	melanin	*Streptomyces olivochromogenes* strain DSM 40451 86%	belactosin A / belactosin C 8%
3.19	ectoine	Multiple *Streptomyces* spp. 100%	ectoine 50%
3.20	NAPAA	*Streptomyces mirabilis* strain OK461 66%	echoside A - E 17%
3.21	ectoine	*Streptomyces griseoviridis* strain F1-27 80%	polyoxypeptin 8%
**3.22**	**NRPS-like**	***Streptomyces canus* strain DSM 40275 24% (unique)**	**avoparcin 5%**
**3.23**	**NRPS**	***Streptomyces* sp. cf386 23% (unique)**	**melanin 71%**
3.24	NRPS,ladderane	*Streptomyces kanamyceticus* strain ATCC 12853 31%	alkylresorcinol 100%
3.25	melanin	*Streptomyces* sp. Act143 100%	cyphomycin 5%
**3.26**	**T3PKS**	***Streptomyces venezuelae* ATCC 10712 26% (unique)**	**foxicins A-D 14%**
3.27	butyrolactone	*Streptomyces* sp. BK308 60%	Putative product
**3.28**	**T1PKS,NRPS-like**	***Streptomyces* sp. BK161 21% (unique)**	**SapB 100%**

Table S5.

Streptomyces sp. LN325			
**Region**	**BGC type**	**Presence in another bacterium**	**Putative product**
1.1	ectoine, oligosaccharide, T1PKS, butyrolactone, NRPS-like, PKS-like, arylpolyene	*Streptomyces* sp. RPA4-2 71%	cyphomycin 43%
1.2	butyrolactone	*Streptomyces subrutilus* strain ATCC 27467 37%	lactonamycin 5%
2.1	T2PKS,indole	*Streptomyces* sp. RPA4-2 100%	spore pigment 83%
2.2	terpene	*Streptomyces* sp. RPA4-2 95%	hopene 84%
2.3	siderophore	*Streptomyces* sp. QMT-28 100%	grincamycin 5%
2.4	hglE-KS,T1PKS	*Streptomyces* sp. RPA4-2 87%	toxoflavin / fervenulin 14%
2.5	terpene	*Streptomyces avermitilis* MA-4680 84%	geosmin 100%
2.6	RiPP-like	*Streptomyces* sp. RPA4-2 100%	-
2.7	siderophore	Multiple *Streptomyces* spp. 100%	-
2.8	terpene	Multiple *Streptomyces* spp. 100%	albaflavenone 100%
2.9	T2PKS,butyrolactone	*Streptomyces* sp. RPA4-2 92%	prejadomycin / rabelomycin / gaudimycin A,C,D / UWM6 35%
2.10	NRPS	*Streptomyces* sp. RPA4-2 87%	-
2.11	siderophore	Multiple *Streptomyces* spp. 100%	desferrioxamin B,E 83%
2.12	melanin	*Streptomyces collinus* Tu 365 90%	istamycin 7%
**2.13**	**RRE-containing**	***Streptomyces lydicus* strain A02 29% (unique)**	**amycolamycin A, B 4%**
2.14	ectoine	Multiple *Streptomyces* spp. 100%	ectoine 100%
2.15	T3PKS	*Streptomyces* sp. RPA4-2 97%	herboxidiene 8%
**2.16**	**NRPS**	***Streptomyces* sp. SAT1 5% (unique)**	**glycopeptidolipid 20%**
2.17	T1PKS	*Streptomyces* sp. RPA4-2 96%	foxicins A-D 48%
2.18	RRE-containing, thiopeptide, LAP	*Streptomyces* sp. RPA4-2 80%	-
2.19	lanthipeptide-class-iii,NRPS,NAPAA	*Streptomyces* sp. QMT-28 95%	stenothricin 13%
2.20	betalactone	*Streptomyces* sp. RPA4-2 92%	-
3.1	RiPP-like	Multiple *Streptomyces* spp. 100%	-
4.1	T3PKS	*Streptomyces* sp. RPA4-2 70%	alkylresorcinol 100%
**4.2**	**terpene**	***Streptomyces* sp. SS 23% (unique)**	**2-methylisoborneol 100%**
6.1	RiPP-like	Multiple *Streptomyces* spp. 100%	informatipeptin 42%
**6.2**	**PKS-like**	***Streptomyces* sp. cf386 50% (unique)**	**sanglifehrin A 6%**

Table S6.

Streptomyces sp. LN499			
**Region**	**BGC type**	**Presence in another bacterium**	**Putative product**
2.1	T2PKS,terpene	*Streptomyces* sp. ADI92-24 80%	spore pigment 83%
2.2	terpene	*Streptomyces aureoverticillatus* strain HN6 41%	kanamycin 1%
3.1	RiPP-like	*Streptomyces* sp. ok210 100%	streptovaricin 4%
3.2	terpene	*Streptomyces atratus* strain SCSIO ZH16 100%	hopene 84%
3.3	RiPP-like	Multiple *Streptomyces* spp. 100%	-
3.4	arylpolyene,ladderane	*Streptomyces* sp. ok210 67%	WS9326 10%
3.5	siderophore	Multiple *Streptomyces* spp. 100%	-
3.6	terpene	Multiple *Streptomyces* spp. 100%	BD-12 17%
3.7	T1PKS,NRPS-like	*Streptomyces* sp. 136MFCol5.1 94%	enduracidin 10%
3.8	T3PKS	*Streptomyces* sp. BK447 36%	naringenin 100%
3.9	siderophore	Multiple *Streptomyces* spp. 100%	desferrioxamin B 100%
3.10	butyrolactone	Multiple *Streptomyces* spp. 100%	-
3.11	ectoine	Multiple *Streptomyces* spp. 100%	ectoine 100%
3.12	terpene	Multiple *Streptomyces* spp. 100%	steffimycin D 19%
3.13	T3PKS,RiPP-like	*Streptomyces* sp. ok210 73%	alkylresorcinol 100%
3.14	NRPS,NAPAA	*Streptomyces* sp. ok210 29%	stenothricin 13%

Table S7.

Streptomyces sp. LN500			
**Region**	**BGC type**	**Presence in another bacterium**	**Putative product**
**2.1**	**T3PKS**	***Streptomyces seoulensis* strain KCTC 9819 16% (unique)**	**violapyrone B 28%**
3.1	RiPP-like	*Streptomyces* sp. ok210 100%	hopene 84%
3.2	terpene	*Streptomyces atratus* strain SCSIO ZH16 100%	-
3.3	RiPP-like	Multiple *Streptomyces* spp. 100%	WS9326 10%
3.4	ladderane	*Streptomyces* sp. ok210 56%	-
3.5	siderophore	Multiple *Streptomyces* spp. 100%	-
3.6	terpene	Multiple *Streptomyces* spp. 100%	BD-12 17%
3.7	T3PKS	*Streptomyces* sp. BK447 36%	naringenin 100%
3.8	siderophore	Multiple *Streptomyces* spp. 100%	desferrioxamin B 100%
3.9	butyrolactone	Multiple *Streptomyces* spp. 100%	-
3.10	ectoine	Multiple *Streptomyces* spp. 100%	ectoine 100%
3.11	terpene	*Streptomyces* sp. ok210 93%	steffimycin D 16%
3.12	T3PKS,RiPP-like	*Streptomyces* sp. ok210 73%	alkylresorcinol 100%
3.13	NRPS,NAPAA	*Streptomyces* sp. ok210 29%	stenothricin 13%
3.14	T2PKS,terpene	*Streptomyces* sp. ADI92-24 80%	spore pigment 83%
3.15	terpene	*Streptomyces aureoverticillatus* strain HN6 41%	kanamycin 1%

Table S8.

Streptomyces sp. LN549			
**Region**	**BGC type**	**Presence in another bacterium**	**Putative product**
**2.1**	**terpene,NRPS**	***Streptomyces* sp. Root55 25% (unique)**	**isorenieratene 100%**
2.2	T2PKS,terpene	*Streptomyces* sp. CB02488 83%	spore pigment 83%
2.3	T3PKS,NRPS	*Streptomyces* sp. SID4920 61%	coelichelin 100%
2.4	NRPS	*Streptomyces seoulensis* strain A01 33%	bacillibactin 100%
2.5	terpene	*Streptomyces* sp. RLB3-17 29%	-
2.6	RiPP-like	*Streptomyces* sp. QL37 50%	-
2.7	NRPS-like,terpene	*Streptomyces* sp. ADI92-24 93%	steffimycin D 19%
2.8	T1PKS,NRPS	*Streptomyces exfoliatus* strain A1013Y 66%	stenothricin 45%
2.9	ectoine	Multiple *Streptomyces* spp. 100%	ectoine 100%
2.10	butyrolactone	*Streptomyces* sp. CB02488 70%	-
2.11	other	*Streptomyces spectabilis* strain NRRL 2792 50%	s56-p1 9%
2.12	terpene	*Streptomyces* sp. CNQ-525 76%	-
2.13	T1PKS,T2PKS	*Streptomyces* sp. ADI92-24 74%	macrotermycins 57%
**2.14**	**NRPS-like,NRPS,T1PKS**	***Streptomyces caeruleatus* strain NRRL B-24802 32% (unique)**	**vazabitide A 6%**
2.15	siderophore	Multiple *Streptomyces* spp. 100%	-
2.16	T2PKS,indole	*Streptomyces* sp. LaPpAH-165 37%	lugdunomycin 66%
2.17	RiPP-like	Multiple *Streptomyces* spp. 100%	-
2.18	NRPS,lanthipeptide-class-ii	*Streptomyces atratus* strain SCSIO 36%	streptolydigin 10%
2.19	terpene	Multiple *Streptomyces* spp. 100%	hopene 76%
**2.20**	**T1PKS,terpene**	***Saccharothrix syringae* strain NRRL B-16468 34% (unique)**	**chlorothricin / deschlorothricin 16%**
2.21	RiPP-like	*Streptomyces* sp. ADI92-24 77%	A54145 5%
**2.22**	**other**	***Streptomyces* sp. NA04227 16% (unique)**	**A-503083 A / A-503083 B / A-503083 E / A-503083 F 7%**

Table S9.

Streptomyces sp. LN590			
**Region**	**BGC type**	**Presence in another bacterium**	**Putative product**
2.1	RiPP-like	*Streptomyces* sp. ok210 85%	-
2.2	terpene	Multiple *Streptomyces* spp. 100%	hopene 84%
2.3	RiPP-like	Multiple *Streptomyces* spp. 100%	-
2.4	arylpolyene,ladderane	*Streptomyces* sp. ok210 67%	WS9326 10%
2.5	siderophore	Multiple *Streptomyces* spp. 100%	-
2.6	terpene	Multiple *Streptomyces* spp. 100%	BD-12 17%
2.7	T1PKS,NRPS-like	*Streptomyces* sp. 136MFCol5.1 94%	leinamycin 4%
2.8	T3PKS	*Streptomyces* sp. BK447 36%	naringenin 100%
2.9	siderophore	Multiple *Streptomyces* spp. 100%	desferrioxamin B 100%
2.10	butyrolactone	Multiple *Streptomyces* spp. 100%	-
2.11	ectoine	Multiple *Streptomyces* spp. 100%	ectoine 100%
2.12	terpene	*Streptomyces* sp. ok210 93%	steffimycin D 16%
2.13	T3PKS,RiPP-like	*Streptomyces* sp. ok210 73%	alkylresorcinol 100%
2.14	NRPS,NAPAA	*Streptomyces* sp. ok210 29%	stenothricin 13%
2.15	T2PKS,terpene	*Streptomyces* sp. ADI92-24 80%	spore pigment 83%
2.16	terpene	*Streptomyces aureoverticillatus* strain HN6 41%	kanamycin 1%

Table S10.

Streptomyces sp. LN699			
**Region**	**BGC type**	**Presence in another bacterium**	**Putative product**
**1.1**	**T1PKS,NRPS-like**	***Streptomyces* sp. SID8369 41% (unique)**	**istamycin 2%**
6.1	T1PKS,butyrolactone	*Streptomyces* sp. 2114.2 69%	coelimycin P1 75%
6.2	LAP	*Streptomyces subrutilus* strain ATCC 27467 92%	-
6.3	T2PKS,butyrolactone, thiopeptide, LAP	*Streptomyces* sp. S1A1-3 53%	prejadomycin / rabelomycin / gaudimycin A,C,D / UWM6 31%
6.4	terpene,PKS-like,butyrolactone, NRPS, NRPS-like	*Streptomyces alboflavus* strain MDJK44 55%	deimino-antipain 66%
6.5	NRPS-like, ladderane, arylpolyene, NRPS, lassopeptide	*Streptomyces peucetius* subsp. *caesius* ATCC 27952 65%	atratumycin 65%
**6.6**	**NRPS**	***Streptomyces viridifaciens* strain ATCC 11989 12% (unique)**	**lavendiol 6%**
9.1	NRPS	*Streptomyces venezuelae* strain ATCC 14583 48%	coelichelin 100%
**9.2**	**lanthipeptide-class-iii**	***Streptomyces pactum* strain ACT12 33% (unique)**	**SapB 100%**
9.3	ectoine	*Streptomyces subrutilus* strain ATCC 27467 66%	ectoine 100%
**9.4**	**other**	***Streptomyces venezuelae* strain ATCC 14583 18% (unique)**	**A-503083 A,B,E,F 5%**
**9.5**	**NRPS**	**- (unique)**	**cadaside A,B 14%**
9.6	terpene	*Streptomyces bingchenggensis* BCW-1 35%	ebelactone 5%
9.7	terpene	*Streptomyces* sp. IMTB 1903 37%	isorenieratene 100%
10.1	RiPP-like	*Streptomyces* sp. CB02400 44%	-
**10.2**	**lanthipeptide-class-iv**	***Streptomyces paludis* strain GSSD-12 33% (unique)**	**venezuelin 100%**
**10.3**	**terpene**	***Streptomyces* sp. CB02400 9% (unique)**	**avermitilol 100%**
10.4	NAPAA,T2PKS	*Streptomyces chartreusis* strain ATCC 14922 34% (unique)	spore pigment 66%
10.5	terpene	*Streptomyces aidingensis* strain CGMCC 52%	-
**10.6**	**NAPAA**	***Streptomyces galilaeus* strain ATCC 14969 13% (unique)**	**MS-271 21%**
10.7	T1PKS,NRPS-like	*Streptomyces prasinus* strain ATCC 13879 47%	prejadomycin / rabelomycin / gaudimycin A,C,D / UWM6 4%
10.8	LAP,thiopeptide,T3PKS	*Streptomyces* sp. DUT11 66%	alkylresorcinol 100%
10.9	siderophore	*Streptomyces* sp. Mg1 72%	salinomycin 4%
10.10	melanin	*Streptomyces* sp. CB02120-2 87%	melanin 28%
10.11	NRPS,T1PKS,NRPS-like	*Streptomyces griseofuscus* strain NG1-7 34%	albachelin 40%
10.12	hglE-KS,T1PKS	*Streptomyces* sp. Sge12 72%	kedarcidin 1%
10.13	terpene	Multiple *Streptomyces* spp. 100%	hopene 61%
10.14	NRPS,terpene	*Streptomyces subrutilus* strain ATCC 27467 43%	toxoflavin / fervenulin 14%
10.15	RiPP-like	*Streptomyces subrutilus* strain ATCC 27467 100%	-
10.16	siderophore	Multiple *Streptomyces* spp. 100%	ficellomycin 3%

10.17	CDPS	*Streptomyces globosus* strain LZH-48 100%	BD-12 10%

Table S11.

***Streptomyces* sp. LN704**			
**Region**	**BGC type**	**Presence in another bacterium**	**Putative product**
2.1	ectoine,oligosaccharide,T1PKS, butyrolactone, PKS-like, NRPS, arylpolyene	*Streptomyces albus* strain DSM 41398 70%	cyphomycin 41%
2.2	RiPP-like	*Streptomyces* sp. RPA4-2 90%	-
2.3	PKS-like	*Streptomyces* sp. cf386 50%	sanglifehrin A 6%
2.4	RiPP-like	Multiple *Streptomyces* spp. 100%	informatipeptin 42%
2.5	T2PKS,indole	*Streptomyces* sp. RPA4-2 100%	spore pigment 75%
2.6	terpene	Multiple *Streptomyces* spp. 100%	hopene 92%
2.7	siderophore	*Streptomyces* sp. QMT-28 100%	grincamycin 5%
2.8	hglE-KS,T1PKS	*Streptomyces* sp. RPA4-2 90%	toxoflavin / fervenulin 14%
2.9	terpene	*Streptomyces avermitilis* MA-4680	geosmin 100%
2.10	RiPP-like	*Streptomyces* sp. RPA4-2 84%	-
2.11	siderophore	Multiple *Streptomyces* spp. 100%	-
2.12	terpene	Multiple *Streptomyces* spp. 100%	albaflavenone 100%
2.13	T2PKS,butyrolactone	*Streptomyces* sp. QMT-28 93%	prejadomycin / rabelomycin / gaudimycin A,C,D / UWM6 35%
2.14	NRPS	*Streptomyces* sp. RPA4-2 95%	-
2.15	siderophore	Multiple *Streptomyces* spp. 100%	desferrioxamin B / desferrioxamine E 83%
2.16	melanin	*Streptomyces olivochromogenes* strain DSM 4045 93%	istamycin 7%
**2.17**	**RRE-containing**	***Streptomyces dioscori* strain A217 12% (unique)**	**amycolamycin A,B 4%**
2.18	ectoine	Multiple *Streptomyces* spp. 100%	ectoine 100%
2.19	NAPAA	*Streptomyces olivochromogenes* strain DSM 40451 73%	-
2.20	T3PKS	*Streptomyces* sp. RPA4-2 97%	herboxidiene 8%
**2.21**	**NRPS**	***Micromonospora mirobrigensis* strain DSM 4483 20% (unique)**	**glycopeptidolipid 20%**
2.22	T1PKS	*Streptomyces* sp. RPA4-2 84%	foxicins A-D 43%
2.23	thiopeptide,LAP	*Streptomyces* sp. RPA4-2 76%	-
2.24	lanthipeptide-class-iii,NRPS,NAPAA	*Streptomyces* sp. QMT-28 95%	stenothricin 13%
2.25	betalactone	*Streptomyces* sp. RPA4-2 92%	-
2.26	melanin	Multiple *Streptomyces* spp. 100%	melanin 71%
**2.27**	**terpene**	***Streptomyces* sp. SS 23% (unique)**	**2-methylisoborneol 100%**
2.28	T3PKS	*Streptomyces* sp. RPA4-2 75%	alkylresorcinol 100%
2.29	butyrolactone	*Streptomyces* sp. RPA4-2 100%	lactonamycin 3%

Table S12.

Streptomyces sp. LN785			
**Region**	**BGC type**	**Presence in another bacterium**	**Putative product**
**1.1**	**NRPS**	***Streptomyces kasugaensis* strain BCRC 12349 31% (unique)**	**ecumicin 10%**
1.2	NRPS	*Streptomyces candidus* strain DSM 40141 54%	s56-p1 11%
1.3	T2PKS,terpene	*Streptomyces* sp. ADI92-24 64%	spore pigment 83%
**1.4**	**NRPS,T3PKS**	***Streptomyces* sp. Tu6071 27% (unique)**	**feglymycin 42%**
**1.5**	**RiPP-like**	***Streptomyces* sp. CB01201 57% (unique)**	**desotamide 9%**
1.6	T3PKS	Streptomyces sp. ok210 55%	alkylresorcinol 100%
1.7	blactam	*Streptomyces finlayi* strain NBSH44 93%	carbapenem MM4550 55%
1.8	T2PKS	*Streptomyces* sp. NBRC 110030 45%	fluostatins M-Q 25%
1.9	terpene	*Streptomyces* sp. ok210 87%	steffimycin D 13%
1.10	ectoine	Multiple *Streptomyces* spp. 100%	ectoine 100%
1.11	butyrolactone	*Streptomyces* sp. 136MFCol5.1 77%	-
1.12	siderophore	*Streptomyces* sp. ok210 88%	desferrioxamin B 100%
1.13	ladderane,T1PKS, phosphonate	*Streptomyces* sp. INR7 45%	amycomicin 100%
1.14	T3PKS	*Streptomyces* sp. BK447 36%	naringenin 100%
1.15	terpene	*Streptomyces* sp. SID4920 64%	-
1.16	siderophore	*Streptomyces* sp. CB02058 88%	-
1.17	RiPP-like	Multiple *Streptomyces* spp. 100%	-
1.18	blactam	*Actinocrispum wychmicini* strain DSM 45934 37%	clavulanic acid 41%
1.19	terpene	Multiple *Streptomyces* spp. 100%	hopene 84%
1.20	RiPP-like	*Streptomyces fulvissimus* DSM 40593 50%	-
1.21	NRPS	*Streptomyces cacaoi* strain OABC16 46%	herboxidiene 5%
**1.22**	**terpene**	***Streptomyces scabiei* 87.22 23% (unique)**	**ebelactone 5%**
**1.23**	**NRPS-like,NRPS**	***Streptomyces* sp. ok210 15% (unique)**	**caniferolide A-D 6%**
**1.24**	**NRPS**	***Streptomyces* sp. CNT302 29% (unique)**	**cephamycin C 36%**

Table S13. Test organism used for antimicrobial activity testing.

Organism	Medium	Growth conditions
Bacillus subtilis DSMZ 10	TSB	28°C, over night
Enterococcus mundtii DSMZ 4840	TSB	37°C, over night
Escherichia coli DH5α	LB	37°C, over night
Kocuria rhizophila DSMZ 348	LB	28°C, over night
Micrococcus luteus DSMZ 1790	TSB	28°C, over night
Pseudomonas putida KT2440	LB	37°C, over night
Saccharomyces cerevisiae BY4743	YPD	28°C, over night
Staphylococcus carnosus DSMZ 20501	TSB	37°C, over night

Table S14. Antimicrobial activity testing results.

Strain	Medium	LN245	LN325	LN499	LN500	LN549	LN590	LN699	LN704	LN785
Bacillus subtilis DSMZ 10	SM17	-	-	-	-	+	-	-	-	-
	MYM	-	-	-	-	+	-	+	-	-
	SG	-	-	-	-	+	-	-	-	-
Enterococcus mundtii DSMZ 4840	SM17	-	-	-	-	-	-	-	-	-
	MYM	-	-	-	-	-	-	-	-	-
	SG	-	-	-	-	-	-	-	-	-
Escherichia coli DH5α	SM17	-	-	-	-	-	-	-	-	-
	MYM	-	-	-	-	-	-	-	-	-
	SG	-	-	-	-	-	-	-	-	-
Kocuria rhizophila DSMZ 348	SM17	-	-	-	-	+	-	-	-	-
	MYM	-	-	-	-	+	-	-	-	-
	SG	-	-	-	-	+	-	-	-	-
Micrococcus luteus DSMZ 1790	SM17	-	-	-	-	+	-	-	-	-
	MYM	-	-	-	-	+	-	-	-	-
	SG	-	-	-	-	+	-	-	-	-
Pseudomonas putida KT2440	SM17	-	-	-	-	-	-	-	-	-
	MYM	-	-	-	-	-	-	-	-	-
	SG	-	-	-	-	-	-	-	-	-
Saccharomyces cerevisiae BY4743	SM17	-	-	-	-	-	-	-	-	-
	MYM	-	-	-	-	-	-	+	-	-
	SG	-	-	-	-	-	-	-	-	-
Staphylococcus carnosus DSMZ 20501	SM17	-	-	-	-	+	-	-	-	-
	MYM	-	-	-	-	+	-	-	-	-
	SG	-	-	-	-	+	-	-	-	-

Table S15. LC-MS results of Edelweiss isolates secondary metabolome.

strain	Rt	*m/z*			sum formula	*m/z*	Δ*m/z*	tentative ID	reference data
		[M+H]+	[M+2H]2+	[M+Na]+	(proposed)	calculated	[ppm]		
LN245	6.8	361.2444			$C_{16}H_{32}N_4O_5$	361.2445	0.4	CAS: 252325-60-3	MS/MS
LN245	9.5	561.3604			$C_{25}H_{48}N_6O_8$	561.3606	0.4	desferrioxamine B	MS/MS
LN245	19.6	435.2160			$C_{19}H_{34}N_2O_7S$	435.2159	-0.2	putatively new NP	
LN245	21.0	449.2315			$C_{20}H_{36}N_2O_7S$	449.2316	0.3	putatively new NP	
LN245	22.6	463.2471			$C_{21}H_{38}N_2O_7S$	463.2472	0.3	putatively new NP	
LN325	9.5	561.3605			$C_{25}H_{48}N_6O_8$	561.3606	0.2	desferrioxamine B	MS/MS
LN325	9.9	376.2076			$C_{16}H_{29}N_3O_7$	376.2078	0.6	putatively new hydroxamate siderophore	
LN325	11.3	562.3082			$C_{24}H_{43}N_5O_{10}$	562.3083	0.2	putatively new hydroxamate siderophore	
LN325	11.9	576.3240			$C_{25}H_{45}N_5O_{10}$	576.3239	-0.1	CAS: 123152-37-4	MS/MS
LN499	11.5	587.3401			$C_{26}H_{46}N_6O_9$	587.3399	-0.3	desmethylenylnocardamine	MS/MS
LN499	11.7	585.3603			$C_{27}H_{48}N_6O_8$	585.3606	0.7	dehydroxynocardamine	MS/MS
LN499	12.3	601.3551			$C_{27}H_{48}N_6O_9$	601.3556	0.7	desferrioxamine E	MS/MS

LN500	9.0	547.3448			$C_{24}H_{46}N_6O_8$	547.3450	0.3	CAS: 1884272-02-9	MS/MS
LN500	9.5	561.3604			$C_{25}H_{48}N_6O_8$	561.3606	0.4	desferrioxamine B	MS/MS
LN500	11.5	587.3401			$C_{26}H_{46}N_6O_9$	587.3399	1.1	desmethylenylnocardamine	MS/MS
LN500	12.3	601.3553			$C_{27}H_{48}N_6O_9$	601.3556	0.4	desferrioxamine E	MS/MS
LN500	13.4	637.3914			$C_{31}H_{52}N_6O_8$	637.3919	0.8	legonoxamine A	MS/MS
LN500	20.7	291.1160			$C_{15}H_{18}N_2O_2S$	291.1162	0.5	CAS: 2101662-05-7	MS/MS
LN549	8.7	517.1815			$C_{25}H_{28}N_2O_{10}$	517.1817	0.4	possibly related to panglimycin E	
LN549	13.5	343.1178			$C_{19}H_{18}O_6$	343.1176	-0.5	panglimycin E	MS/MS
LN549	13.7	401.1227			$C_{21}H_{20}O_8$	401.1231	1.1	daidzein-rhamnoside	
LN549	14.5	327.1225			$C_{19}H_{18}O_5$	327.1227	0.8	possible cangumycin isomer	
LN549	17.2			349.1046	$C_{19}H_{18}O_5$	349.1046	0.2	possible cangumycin isomer	
LN549	18.0	531.1860			$C_{27}H_{30}O_{11}$	531.1861	0.2	putatively new glycosylated genistein	
LN549	18.4	369.1330			$C_{21}H_{20}O_6$	369.1333	0.8	putatively new glycosylated daidzein	
LN549	21.9	420.2530			$C_{27}H_{33}NO_3$	420.2533	0.8	bombyxamycin A isomer	
LN549	23.0	254.0810			$C_{15}H_{11}NO_3$	254.0812	0.7	utahmycin A	
LN549	23.4	432.2166			$C_{27}H_{29}NO_4$	432.2169	0.7	piceamycin	UV

LN549	23.4	420.2529			$C_{27}H_{33}NO_3$	420.2533	1.1	possible bombyxamycin A isomer	
LN549	24.0	307.0963		329.0781	$C_{19}H_{14}O_4$	307.0965	0.6	possible ochromycinone isomer	
LN549	24.9	307.0962			$C_{19}H_{14}O_4$	307.0965	0.9	ochromycinone	
LN549	27.2	309.1124		331.0941	$C_{19}H_{16}O_4$	331.0941	0.0	emycin A	
LN590	9.0	547.3448			$C_{24}H_{46}N_6O_8$	547.3450	0.3	CAS: 1884272-02-9	MS/MS
LN590	9.5	561.3599			$C_{25}H_{48}N_6O_8$	561.3606	1.3	desferrioxamine B	MS/MS
LN590	11.5	587.3395			$C_{26}H_{46}N_6O_9$	587.3399	0.7	desmethylenylnocardamine	MS/MS
LN590	12.3	601.3551			$C_{27}H_{48}N_6O_9$	601.3556	0.7	desferrioxamine E	MS/MS
LN590	13.4	637.3917			$C_{31}H_{52}N_6O_8$	637.3919	0.4	legonoxamine A	MS/MS
LN590	20.6	291.1160			$C_{15}H_{18}N_2O_2S$	291.1162	0.6	CAS: 2101662-05-7	MS/MS
LN699	5.0	426.1252			$C_{16}H_{19}N_5O_9$	426.1256	0.9	aureonucleomycin	UV
LN699	6.9	238.1660			$C_{11}H_{19}N_5O$	238.1662	0.9	argvalin	
LN699	8.4	252.1816			$C_{12}H_{21}N_5O$	252.1819	1.2	arglecin	
LN699	8.6	366.1770			$C_{16}H_{23}N_5O_5$	366.1772	0.5	metabolite KF77-AG6	
LN699	9.0-12.0		303.1795		$C_{27}H_{44}N_{10}O_6$	303.1795	0.1	antipain	
LN699	9.7		304.1871		$C_{27}H_{46}N_{10}O_6$	304.1874	0.8	reduced antipain	

LN699	10.0-13.6	427.3025			$C_{20}H_{38}N_6O_4$	427.3027	0.7	leupeptin	MS/MS
LN699	10.6-14.4	441.3183			$C_{21}H_{40}N_6O_4$	441.3184	0.3	leupeptin Pr-LL	MS/MS
LN699	11.6	429.3184			$C_{20}H_{40}N_6O_4$	429.3184	0.0	reduced leupeptin	
LN699	11.7	252.1704			$C_{13}H_{21}N_3O_2$	252.1707	1.0	streptopyrazinone A or D	
LN699	12.1	464.2614			$C_{21}H_{33}N_7O_5$	464.2616	0.4	unknown congener	
LN699	12.2	252.1702			$C_{13}H_{21}N_3O_2$	252.1707	1.7	streptopyrazinone A or D	
LN699	12.8	443.3336			$C_{21}H_{42}N_6O_4$	443.3340	0.9	reduced leupeptin Pr-LL	
LN699	15.2-17.0	596.3188			$C_{30}H_{41}N_7O_6$	596.3191	0.5	MAPI	
LN699	15.9	598.3344			$C_{30}H_{43}N_7O_6$	598.3348	0.6	Mer-N5075-A (red. MAPI)	MS/MS
LN699	15.9	612.3138			$C_{30}H_{41}N_7O_7$	612.3140	0.4	oxidized MAPI	
LN699	16.1	727.3776	364.1924		$C_{35}H_{50}N_8O_9$	727.3774	-0.4	unknown congener	
LN699	18.3	229.1333			$C_{14}H_{16}N_2O$	229.1335	0.9	phevalin	MS/MS and UV
LN704	9.0	547.3451			$C_{24}H_{46}N_6O_8$	547.3450	-0.1	CAS: 1884272-02-9	MS/MS
LN704	9.5	561.3606			$C_{25}H_{48}N_6O_8$	561.3606	0.0	desferrioxamine B	MS/MS
LN704	9.9	376.2073			$C_{16}H_{29}N_3O_7$	376.2078	1.4	putatively new hydroxamate siderophore	
LN704	11.3	562.3077			$C_{24}H_{43}N_5O_{10}$	562.3083	1.1	putatively new hydroxamate siderophore	

LN704	11.3	533.2811			$C_{23}H_{40}N_4O_{10}$	533.2817	1.1	desferrioxamine B congener	
LN704	11.5	560.3283			$C_{25}H_{45}N_5O_9$	560.3290	1.3	desferrioxamine B congener	
LN704	11.7	585.3597			$C_{27}H_{48}N_6O_8$	585.3606	1.5	dehydroxynocardamine	MS/MS
LN704	11.7	532.2970			$C_{23}H_{41}N_5O_9$	532.2977	1.3	desferrioxamine B congener	
LN704	12.0	576.3237			$C_{25}H_{45}N_5O_{10}$	576.3239	0.3	CAS: 123152-37-4	MS/MS
LN704	12.3	601.3549			$C_{27}H_{48}N_6O_9$	601.3556	1.0	desferrioxamine E	MS/MS
LN785	9.0	547.3448			$C_{24}H_{46}N_6O_8$	547.3450	0.4	CAS: 1884272-02-9	MS/MS
LN785	9.4	561.3605			$C_{25}H_{48}N_6O_8$	561.3606	0.3	desferrioxamine B	MS/MS
LN785	11.5	587.3401			$C_{26}H_{46}N_6O_9$	587.3399	-0.3	desmethylenylnocardamine	MS/MS
LN785	12.3	601.3559			$C_{27}H_{48}N_6O_9$	601.3556	-0.6	desferrioxamine E	MS/MS
LN785	12.4	354.0969			$C_{19}H_{15}NO_6$	354.0972	1.0	possible congener of WS-5995 C	
LN785	13.4	637.3919			$C_{31}H_{52}N_6O_8$	637.3919	0.0	legonoxamine A	MS/MS
LN785	18.5	355.0812			$C_{19}H_{14}O_7$	355.0812	0.2	WS-5995 C	MS/MS
